# Procedural Versus Baked Textures in Path-Traced Rendering: A Controlled Comparison of Render Time and Memory Footprint in Blender Cycles

**DOI:** 10.3390/jimaging12100471

**Published:** 2026-09-27

**Authors:** Ilija Biškup, Matija Grašić, Andrija Bernik

**Affiliations:** Department of Multimedia, University North, Ulica 104. Brigade 3, 42000 Varaždin, Croatia; ilija.biskup@gmail.com (I.B.); abernik@unin.hr (A.B.)

**Keywords:** procedural textures, texture baking, path tracing, Blender Cycles, render performance, shader complexity, ambient occlusion, memory footprint

## Abstract

Procedural textures trade image storage for runtime shader evaluation, whereas baked bitmaps replace repeated procedural computation with stored image data. Controlled path-tracing comparisons that isolate this representation choice remain limited. This study compares four procedural Base Color graphs with their $2048^{2}$ baked bitmap counterparts in Blender Cycles 5.2 on a GIGABYTE NVIDIA GTX 1050 Ti (GIGA-BYTE Technology Co., Ltd., New Taipei City, Taiwan) while holding geometry, lighting, camera, sampling and BSDF parameters constant. Three texture graphs—checker, noise and multifractal—were examined in a predefined ordering by computational demand, while ambient occlusion was analyzed separately as a ray-traced effect. The primary experiment comprised 480 renders, of which 360 were analyzed using block means as the inferential unit. Block-level models showed that the procedural checker was 0.65% faster than its baked counterpart, whereas the bitmap representation was 1.73% faster for noise and 3.53% faster for multifractal; all four primary representation effects had 95% confidence intervals excluding zero after Holm adjustment. Baking ambient occlusion reduced render time by 67.74% under the tested configuration. The bitmap branch increased Cycles-reported internal peak memory by exactly 16 MiB at $2048^{2}$, while resolution tests produced increments of 1, 4 and 64 MiB at $512^{2}$, $1024^{2}$ and $4096^{2}$, respectively. Because the $2048^{2}$ baked multifractal showed lower visual agreement with its procedural source, a targeted $4096^{2}$ follow-up comprising a further 120 renders, of which 90 were analyzed, was conducted. Increasing the multifractal bake resolution improved SSIM from 0.886 to 0.936, increased the image-texture footprint from 16 to 64 MiB, and reduced the bitmap render-time advantage from 3.53% to 1.73%. Across the three primary texture graphs, the bitmap advantage increased along the predefined ordering by computational demand; however, computational demand was not isolated from differences in algorithm, coordinate system, and spatial structure. The results therefore demonstrate configuration-specific trade-offs among render time, image-memory footprint, and representation fidelity rather than a universal preference for either representation.

## 1. Introduction

Texture mapping has been a fundamental component of computer graphics since early methods introduced detailed surface appearance without requiring equivalent geometric complexity [1,2]. Subsequent work systematized texture-mapping techniques and identified parameterization, sampling, and filtering as central technical challenges [3], while shade trees established a programmable framework in which surface appearance could be computed rather than stored explicitly [4]. These developments underpin two representation strategies that remain central to rendering: bitmap textures, which store sampled appearance data, and procedural textures, which generate appearance algorithmically during shading.

Procedural texturing uses mathematical functions to generate complex surface detail from compact descriptions. Perlin introduced gradient-noise-based procedural texture generation [5] and later refined its formulation [6], while cellular basis functions [7] and fractal constructions [8] extended the range of available procedural patterns. These methods and their properties have since been reviewed comprehensively [9]. A principal advantage of procedural representations is that complex, resolution-independent appearance can be generated without storing a corresponding high-resolution image; the trade-off is that the appearance must instead be evaluated computationally during shading [10].

The resulting computation–memory trade-off is important in modern rendering. Real-time systems depend heavily on efficient texture sampling, filtering, caching, and memory bandwidth [11], and production pipelines therefore commonly bake procedural or lighting information into textures to reduce runtime cost [12,13]. Offline path tracing tolerates greater shader complexity, but shader evaluation, texture access, memory use, and Monte Carlo sampling still contribute to total rendering cost [14]. Because baking can also alter the represented signal, visual agreement must be considered alongside performance; SSIM [15] and FLIP [16] provide complementary measures for assessing structural and perceptually relevant differences between rendered images.

The computational cost of procedural shading has been recognized since early interactive graphics. Real-time procedural texturing was demonstrated by Rhoades et al. [17], followed by shading systems that mapped procedural descriptions onto specialized [18] and programmable graphics hardware [19]. Carr and Hart later demonstrated a directly related strategy in which procedural solid textures were converted into texture atlases for later conventional texture sampling [20]. Bitmap representations introduce different costs: compressed texture rendering [21], production systems such as Ptex [22] and alternative texture-mapping representations [23] all illustrate the interaction between storage, sampling, filtering and runtime performance. Reviews of procedural texture generation similarly emphasize the exchange of explicit image storage for runtime computation [24].

This trade-off remains relevant in contemporary path tracing. Procedural noise evaluation can represent a meaningful computational cost in Monte Carlo rendering [25], while recent work on stochastic and collaborative texture filtering shows that bitmap sampling, decompression, and filtering also impose non-negligible costs on modern GPUs [26,27]. Recent research on selective caching in procedural texture graphs further demonstrates that the balance between runtime evaluation, precomputation, and storage depends on the characteristics of the rendering workload [28]. Consequently, neither representation can be assumed to be universally cheaper, particularly when procedural graphs range from simple analytical patterns to multi-octave noise.

Precomputation can be especially beneficial when the evaluated quantity requires geometric queries. Ambient occlusion can be precomputed and reconstructed at lower runtime cost [29], and similar computation-for-storage strategies are used in precomputed global illumination [30] and production baking systems such as UberBake [31]. The same principle is relevant to modern offline production rendering, where path tracing has become practical at feature-film scale [32]. Published production-rendering architectures, including RenderMan [33] and Arnold [34], must process complex geometry, materials, textures, and large numbers of shading evaluations, making even relatively small per-evaluation costs potentially relevant.

Despite this extensive literature, previous work has primarily addressed procedural-function design, texture representation, shading optimization, filtering, and application-specific precomputation. Direct controlled evidence comparing a procedural material with its baked bitmap counterpart under otherwise identical path-tracing conditions, while measuring both render time and memory footprint, remains limited. The present study addresses this narrower question in Blender Cycles by comparing procedural Base Color graphs with baked bitmap representations while holding geometry, lighting, camera, sampling settings, and BSDF parameters constant. Three procedural texture graphs are examined in a predefined ordering by computational demand, ambient occlusion is treated separately as a ray-traced effect, and the visual agreement between each procedural representation and its baked counterpart is evaluated independently.

The study addresses three research questions. RQ1 asks whether a measurable and repeatable render-time difference exists between procedural and baked representations. RQ2 examines how this difference varies across the three tested procedural shader graphs in their predefined ordering by computational demand. RQ3 examines how the two representations differ in memory footprint. The corresponding hypotheses are:

**Hypothesis** **1** (H1)**.** *Repeatable render-time difference exists.*

**Hypothesis** **2** (H2)**.** *The bitmap advantage increases across the tested graph ordering, with the procedural representation expected to be less costly for the simplest pattern and the bitmap representation increasingly favorable for the more demanding graphs.*

**Hypothesis** **3** (H3)**.** *The procedural representation has the smaller, resolution-independent image-memory footprint. Ambient occlusion is examined separately because it performs scene-dependent ray queries rather than representing another level of arithmetic texture complexity.*

## 2. Materials and Methods

### 2.1. Hardware and Software

All measurements were taken on one desktop workstation, which was not used for any other task while a batch was running. The configuration is given in Table 1, in enough detail that the measurement can be reproduced on comparable hardware.

The workstation contained 16 GB of system RAM as two 8 GB DDR4 modules, both operating at 2400 MT/s, and used NVIDIA driver version 582.28, which reports CUDA 13.0. The operating system was Windows 10 Home, version 22H2, build 19045. The absolute times reported here therefore describe an unmodified desktop configuration rather than a tuned one. Because the plan was the same in every condition, it cannot bias the comparison between representations, although a machine on a high-performance plan would be expected to produce somewhat shorter absolute times. The GTX 1050 Ti used for the primary experiment is a pre-RTX Pascal-generation GPU, and the measurements were performed with the CUDA compute backend. The primary benchmark therefore does not evaluate the dedicated hardware ray-tracing acceleration available on newer RTX-class GPUs through the OptiX backend; OptiX was not used in the present experiment. This hardware and backend choice is treated as part of the experimental configuration rather than as a representative model of all contemporary Cycles systems.

Every batch was run in a single uninterrupted session with no other GPU activity, as described in Section 2.8.

### 2.2. Render Configuration

All render settings were written programmatically to the .blend files before measurement and remained unchanged across conditions (Table 2). Sampling was fixed and non-adaptive so that every render used the same sampling budget, and denoising was disabled to prevent post-processing from obscuring differences in rendering cost. Persistent Data was also disabled so that render-time scene data were rebuilt for each render rather than retained between successive renders; the implications and limitations of this setting are described below.

The maximum total path depth was set to 12, while diffuse paths were limited to four bounces. Because the shared BSDF is effectively diffuse, with Metallic 0, Roughness 1 and Specular IOR Level 0 as described in Section 2.4, the diffuse limit is the relevant constraint, and the total depth limit of 12 is not reached. Minimum Light Bounces was set to 12 to suppress stochastic early path termination within the applicable path-depth limits. In Cycles, paths may otherwise terminate early when their remaining contribution becomes sufficiently small, and the frequency of such termination can depend on surface brightness. Because the tested materials differ in brightness and a baked representation is not an exact numerical reproduction of its procedural source, uncontrolled early termination could introduce a timing difference unrelated to shader-evaluation cost. Increasing Minimum Light Bounces therefore reduced this potential source of variation, at the expense of longer absolute render times.

Adaptive sampling was explicitly disabled, so all 256 samples are taken at every pixel irrespective of the estimated noise there. This matters for the comparison: a baked map and the pattern it replaces differ slightly in local contrast, and with adaptive sampling active the two branches would receive different numbers of samples, so the measurement would no longer be of equal work. Denoising was disabled for the same reason, so that no post-process can absorb a difference in sampling cost.

Texture Limit was left at Off, so no resolution downscaling was applied to the baked maps. The Cycles Texture Cache was not treated as an experimental factor in this study; consequently, the memory results reported below describe the measured behavior of the tested Blender configuration rather than a general texture-caching model. Automatic tiling was enabled with a tile size of 2048 px, which at a render resolution of 1000 × 1000 px means each image is always rendered as a single tile. The resolution percentage was 100, so the stated pixel dimensions are the dimensions actually rendered, and sampling was fixed rather than adaptive, so the sample count in Table 2 is the count taken at every pixel.

Persistent Data was disabled. With Persistent Data off, Blender releases the render-time scene data after each render and rebuilds it for the next, so the acceleration structure and the device-side scene are not carried from one render to the next. What does persist is whatever lives outside that data for the lifetime of the process and the driver, in particular the compiled kernel, the driver’s own caches, and the image already loaded into Blender’s data. Section 2.8 describes what happened between blocks, and the three discarded renders at the start of every block exist precisely because that residual state cannot be eliminated by a setting.

Three further settings were left at their Blender defaults rather than adjusted for this study, and are recorded here so that the light-transport configuration is fully specified. Clamp Indirect was active at 10.0, which limits the intensity any single indirect sample may contribute; reflective and refractive caustics remained enabled; and the Light Threshold remained at 0.01. The last of these is worth separating from Minimum Light Bounces, because the two are different mechanisms: raising the minimum suppresses Russian-roulette termination of a path, whereas the Light Threshold discards light samples whose contribution falls below a cut-off. Suppressing the first therefore does not disable the second, and a residual dependence of sampling work on surface brightness remains, which is one reason the Base Color floor described in Section 2.4 is reported as a reduction rather than an elimination. Both are identical in every condition, and with a purely diffuse BSDF lit by a low-dynamic-range World gradient neither is expected to have any effect on the reported times; they are disclosed because a reader opening the scene files would otherwise find them undocumented.

### 2.3. Scene, Camera, and Lighting

The test object is the Suzanne mesh carrying two levels of Catmull–Clark subdivision, applied destructively to the mesh data, which yields 7958 vertices and 7872 faces, all quadrilaterals. Because the subdivision is applied rather than left as a live modifier, the object carries no modifiers at all, and none is evaluated at render time. The object is smooth-shaded and has no custom normals. It carries a single UV layer, the built-in map named UVMap, which is both the active layer and the render-active layer and was not edited; this is the parameterization into which every material was baked. The concave regions and self-shadowing of this mesh are necessary for the ambient-occlusion condition to have anything to compute.

The object transform is location (0.000, −0.172, 1.043) m, XYZ Euler rotation (−2.243°, −0.467°, 80.833°) and a uniform Object Scale of 1.18614. The scale was deliberately left unapplied so that the scene files remain exactly as measured; the resulting world-space bounding box is 3.151 × 1.899 × 2.267 m. Since the same object data, transform, and unapplied scale are present in all eight scene files, the scale cannot contribute any difference between conditions.

The camera is a 50 mm perspective lens on a 36 × 24 mm sensor with sensor fit Auto, giving a horizontal field of view of 39.60°, a clipping range of 0.1 m to 1000 m and depth of field disabled. It sits at (4.475, −0.117, 1.026) m with XYZ Euler rotation (90°, 0°, 90°), so it looks along the negative X axis towards the object. The camera is fixed for all measurements and is byte-for-byte identical across the eight scene files.

Illumination comes solely from a node-based World shader, which is likewise identical in all eight files. A Texture Coordinate node supplies generated coordinates to a Mapping node in Point mode, which applies no translation and no scale but rotates the vector by 90° about the Y axis; the rotated vector drives a Gradient Texture in Linear mode, whose output feeds the Color input of a Background node with Strength 2.0, which in turn feeds the World Output. The rotation is what turns the gradient’s native horizontal sweep into the vertical luminance gradient used here. The scene contains no light objects and no HDRI, which removes uncontrolled specular contributions as a source of variance.

Film transparency was enabled during all timing measurements, so the background is empty in the rendered image while the World shader still lights the object. For Figure 1 only, the world background was retained to illustrate the lighting setup; those stills were produced from the same scene files by a separate script at a reduced resolution of 800 × 800 px and are illustrative rather than measurements. Both branches of every material are produced from the same template scene, so geometry, camera, and lighting are identical between them; the only difference between any two of the eight files is the material assigned to the object’s single material slot.

### 2.4. Standardized Shading Model

Both branches of every material use the same Principled BSDF with Metallic 0, Roughness 1 and Specular IOR Level 0. Only the source of the Base Color input differs: a procedural node graph in one branch, an Image Texture node reading the baked map in the other. No normal, roughness or displacement maps are used in either branch.

These three values were chosen deliberately. Metallic 0 removes the metallic lobe, Roughness 1 makes the remaining reflection maximally diffuse, and Specular IOR Level 0 removes the dielectric specular lobe, so the surface response is as close to purely Lambertian as the Principled BSDF permits. The purpose is to suppress reflective and view-dependent behavior, which carries a shading cost of its own that varies with viewing geometry and local surface orientation and would be superimposed on the quantity under study. What remains is a shading cost dominated by evaluation of the Base Color chain, which is the variable the experiment manipulates.

No part of any material is allowed to reach pure black. The darkest value produced by every Base Color chain is floored at 0.05 rather than 0. A perfectly black diffuse surface absorbs all the light that reaches it, so a path ending there carries nothing onwards and costs nothing further to trace. The share of the surface that is dark differs between the four materials, and differs slightly between a procedural pattern and its baked copy, so permitting pure black would let the amount of tracing work depend on how dark the pattern happens to look rather than on the cost of computing it. Flooring the darkest value at 0.05 reduced, but did not completely eliminate the possibility that differences in surface brightness influenced path termination. The four materials still differ in mean brightness and in the spatial distribution of their light and dark regions, so the amount of light transport is not equal across the eight cells; it is confined to a narrower range than it would otherwise occupy. Together with the fixed bounce depth described in Section 2.2, the floor limits how far that residual difference can propagate, and the comparison that carries the argument is in every case between the two branches of one material, where the pattern, and therefore the brightness distribution, is nominally the same.

### 2.5. Test Materials

Four materials were used in two categories. Three procedural shader graphs were tested in a predefined ordering by procedural evaluation demand. Ambient occlusion was treated as a separate case because it operates through a different mechanism: the node performs scene-dependent ray queries rather than evaluating an arithmetic texture pattern (Table 3).

The first three materials were selected to provide a predefined ordering of procedural evaluation demand rather than a controlled one-dimensional scale of shader complexity. The checker pattern reduces to a parity test on the scaled texture coordinate and therefore represents the simplest tested graph. The noise material evaluates fractal Brownian motion with Detail 8, while the multifractal material evaluates fifteen octaves and additionally applies a coordinate distortion of 0.5 involving further noise evaluations.

This ordering is used only as a descriptive organization of the three tested graphs. Computational demand was not manipulated independently: the materials also differ in algorithm, coordinate system, spatial structure, and resulting surface-brightness distribution. The checker samples the UV layer, whereas noise and the multifractal sample Generated object space. Consequently, the spacing between the three conditions is not a measured quantity, and differences in render-time behavior across them cannot be attributed uniquely to computational demand.

All four materials are sample color only, so the comparison is confined to the Base Color channel throughout. Figure 1 shows the four materials as rendered on the test object, and Figure 2 places each procedural branch beside its baked counterpart.

Table 3 lists the node chain of each procedural branch. Every chain terminates in the Base Color input of the shared Principled BSDF, and node names and parameters are those of Blender 5.2, in which the Color Ramp node is ShaderNodeValToRGB and the Ambient Occlusion node is ShaderNodeAmbientOcclusion. Node count is not a comparable measure of cost across materials.

The coordinate column is marked n/a for ambient occlusion because the Ambient Occlusion node samples no texture coordinate: it casts rays into the scene over a radius expressed in Blender units. Its distance of 1.0 should therefore be read against the object’s world-space bounding box of 3.151 × 1.899 × 2.267 m given in Section 2.3, which places the radius at roughly one-third of the object’s largest dimension. The node graph of each procedural material is reproduced as a screenshot of the Blender Shader Editor in Appendix A. The scene files remain the authoritative record of every parameter and are available as described in the Data Availability Statement.

Base Color values are given in linear scene-referred units.

### 2.6. Texture Baking

Each procedural material was baked into a single 2048 × 2048 map using the DIFFUSE bake pass in Blender 5.2.0 LTS pass restricted to color, with direct and indirect lighting contributions excluded, 32 bake samples, a 16 px margin, and lossless PNG output. The same procedure was applied to all four materials, including AO, whose node output is treated as color. A $2048^{2}$ bake resolution was selected to reduce the risk of undersampling relative to the $1000^{2}$ output image. Whether that risk was in fact eliminated is not something the resolution alone can establish, and it is measured in Section 2.6. The same protocol was repeated at 512, 1024, and 4096 to quantify how the stored footprint scales with resolution, reported in Section 3.3.

The baked maps are written and read as 8-bit sRGB, which is the standard interchange format for color maps in Blender, while the render itself is displayed through the AgX view transform. Both branches pass through the same view transform, so the transform cannot favor either one. Baking in UV space does mean that the noise and multifractal materials, whose procedural branches evaluate in Generated object space, are sampled in a different parameterization once baked. This is inherent to baking rather than a deviation from the protocol.

Every bitmap branch reads its map through an Image Texture node with identical settings: Interpolation Linear, Projection Flat, Extension Repeat, Color Space sRGB, and Alpha Mode Straight. The maps are stored as 8-bit PNG and carry no alpha channel. The Repeat extension mode is never exercised, because the object’s UV layer lies within the unit square, spanning 0.0001 to 0.9999 in u and 0.0094 to 0.9851 in v.

#### Perceptual Equivalence of the Two Branches

The comparison of render times presumes that a baked map and the procedural pattern it replaces look the same. That presumption was tested rather than assumed. For each material the Diffuse Color pass was rendered for both branches at the measurement resolution of 1000 × 1000 px, at 64 samples, and written as linear 32-bit OpenEXR. This pass records the Base Color reaching the surface before any light transport, so it contains neither illumination nor path-tracing noise and isolates the texture itself, which is the quantity in question. The two images were then encoded to sRGB and compared using SSIM [15] and FLIP [16]. Both metrics were evaluated over the pixels the object covers, taken as those where the albedo of either branch exceeds 0.002 in any channel; the threshold separates the object from the transparent background unambiguously, because no Base Color chain is permitted below 0.05. The background is excluded because a large constant region would inflate SSIM and deflate mean FLIP without carrying any information.

Table 4 reports the outcome, and Figure 3 shows where the two branches differ. Three of the four materials agree closely. Checker, noise, and ambient occlusion reach SSIM between 0.998 and 0.999 with a mean FLIP between 0.007 and 0.016, and their error maps are nearly empty. For checker, the residual is confined to the boundaries between light and dark squares, where a sampled map cannot reproduce a step edge exactly; for ambient occlusion, it is confined to thin arcs at the rims of the eye sockets, where the occlusion gradient is steepest. The multifractal is the exception, at SSIM 0.886 and mean FLIP 0.083.

The difference in the multifractal noise texture is a diffuse loss of fine detail spread across the whole surface rather than a seam or a registration error. This is the expected behavior of a fifteen-octave pattern at Scale 2, which carries spatial frequencies finer than a $2048^{2}$ map can represent over this object’s UV layout. Baking the same material at 1024 and at 4096 and repeating the comparison gives SSIM 0.743 and 0.936, respectively, against 0.886 at 2048. Agreement therefore improves monotonically with bake resolution.

The multifractal timing result should not be interpreted as an equal-fidelity performance comparison. At the common $2048^{2}$ bake resolution, the bitmap representation was 3.53% faster than the procedural branch, but it also showed substantially lower visual agreement with its procedural source than the other tested materials (SSIM = 0.886; mean FLIP = 0.083). The 3.53% value therefore quantifies the performance difference between the two representations at the fixed $2048^{2}$ bake resolution, not the performance advantage of a perceptually equivalent substitute. Because increasing bake resolution simultaneously changes visual fidelity and memory cost, the performance–fidelity trade-off of the multifractal case cannot be inferred from the $2048^{2}$ timing result alone.

Increasing the bake resolution improved multifractal agreement from SSIM 0.743 at $1024^{2}$ to 0.886 at $2048^{2}$ and 0.936 at $4096^{2}$, although even the $4096^{2}$ result remained less similar than those of the other three materials. The primary comparison therefore retains a common $2048^{2}$ bake resolution for all four materials so that the main timing and memory comparisons share the same bitmap resolution. To examine the multifractal performance–fidelity trade-off at the higher-fidelity bake resolution, a targeted follow-up timing experiment was additionally conducted with the $4096^{2}$ multifractal bitmap. The procedural multifractal branch was re-measured in the same follow-up sessions rather than being compared with historical timing values. The follow-up used the same two-part order-balanced protocol as the primary experiment: Part 1 followed procedural–bitmap–procedural–bitmap–procedural order and Part 2 used the reverse order, with three warm-up renders followed by nine analyzed renders in each block. The follow-up therefore comprised 120 renders in total, of which 90 were included in the analysis. All render settings and scene parameters were held constant; only the bitmap bake resolution differed from the primary multifractal comparison.

### 2.7. Instrumentation

Blender does not emit structured per-render metrics, so a measurement script was embedded in every .blend file as a registered text block. It writes one JSON report, one human-readable report and one row of a cumulative tab-separated log for every render, the log carrying fourteen columns.

Timing uses the Python 3.14.15 time.perf_counter() function and Blender 5.2.0 LTS render handlers. A counter is taken in render_init, a second in render_pre, and a third when render_post fires. Pure sampling time is the interval from render_pre to render_post. Total render time is the interval from render_init to render_post. Setup time is not measured independently but derived by subtraction, as total minus pure sampling time, so it covers only what Blender does between render_init and render_pre and nothing else. A fourth handler, render_cancel, writes the same record with a canceled flag; no render reported in this study was canceled. GPU temperature and clock are read through nvidia-smi after the third counter is taken, so that the subprocess call cannot fall inside any reported interval.

Two memory quantities are reported, and they represent different concepts. The first is an internal renderer metric reported by Cycles and is referred to throughout as Cycles-reported internal peak memory. It is obtained by matching the “Mem:” field of the status strings passed by Cycles to the render_stats handler and retaining the largest value reported during each render, with values converted from gigabytes where necessary. This quantity reflects Cycles’ internal accounting of allocations associated with the render, including geometry, acceleration structures, kernel data, and textures. Cycles reports it as a single value and does not separate device from host allocation. It must therefore not be interpreted as a direct measurement of physical GPU VRAM consumption or total system RAM usage, and the values reported below should not be compared directly with measurements from tools such as nvidia-smi or the Windows memory monitor. The second quantity is calculated rather than measured: the image-texture footprint is the arithmetic product of image width, height, channel count, and bytes per channel, summed over the images loaded in the file, using one byte per channel for integer images and four bytes per channel for floating-point images. This calculated footprint is exact by construction and is zero in every procedural branch, making it useful for isolating the storage contribution of the baked image maps.

GPU clock locking is not supported on the card used, so clock and temperature were logged for monitoring rather than for control, and their observed ranges are reported in Section 3.1.

Setup time as defined above is effectively zero. Across the 480 renders of the primary experiment, it was exactly zero in 476 and at most 0.2 ms in the remaining four, and the largest difference between total render time and pure sampling time in that primary dataset is 0.1 ms. This holds equally for the first render of every block, which follows a file open. The derived setup figure therefore does not capture acceleration-structure construction or kernel compilation, and these logs do not establish where that work is performed: it may occur after render_pre, which would place it inside the interval reported as pure sampling time, or during the file open before render_init fires. No claim is made either way, and demonstrating it would require a profiler rather than these handlers.

The logs do record a start-of-session effect, although it did not recur in the sessions reported here. In a session of ambient occlusion that was later superseded, for the reason given in Section 2.8, the first render took 42.78 s against a mean of 38.97 s for the measured renders of that block, 9.8 % higher, and no elevation of comparable size appeared at the first render of any later block. In the eight primary-experiment sessions, the three warm-up renders of each block agree with the measured renders that follow them to within about 1%. The three discarded renders at the start of each block absorb that effect, but they are not claimed to isolate any particular one-time cost. Total render time equals pure sampling time to within the resolution of the timer, so only pure sampling time is reported in what follows.

### 2.8. Measurement Protocol

Each material was evaluated in two parts conducted as separate sessions on different occasions. Together, the two parts form a single order-balanced experiment. Part 1 comprised five consecutive blocks in the order procedural–bitmap–procedural–bitmap–procedural, whereas Part 2 used the reverse order, bitmap–procedural–bitmap–procedural–bitmap. Both parts were retained and analyzed, and agreement between their representation effects was used as a robustness check. Each block comprised twelve renders: three warm-up renders that were logged but excluded from all reported analyses, followed by nine measured renders included in the analysis. Consecutive blocks were separated by a 20 s pause. Alternating procedural and bitmap conditions within each session reduces the risk that a monotonic change in machine state is confounded with representation, while reversing the order in Part 2 counterbalances which representation occupies the first and last block positions.

A single five-block alternation cannot balance the branches, because it necessarily gives three blocks to whichever branch starts and ends it and two to the other. Part 1 alone therefore yields 27 measured procedural renders against 18 bitmap, with the procedural branch always first, and part 2 the reverse. Only across the pair is the design balanced: each branch then receives five blocks and 45 measured renders, and each branch occupies the first position of one part and the last position of the other. Each of the four primary materials contributes 120 renders, of which 90 are analyzed, giving 480 renders in total in the primary experiment, 360 of which are analyzed.

The seed formula is identical in both parts: the Cycles sampling seed is the block number multiplied by one hundred, plus the index of the render within the block. Because only the block order is reversed, every seed drawn by the procedural branch in Part 1 is drawn by the bitmap branch in Part 2, and vice versa. The two branches are therefore matched seed for seed across the pair, so each measured procedural render has a bitmap counterpart that used the identical sampling pattern.

Each block opened its own .blend file using wm.open_mainfile and performed all twelve renders while that file remained open. The procedural and bitmap branches were stored in separate scene files rather than being edited within a shared file, so every block boundary involved loading the corresponding scene file. Blender remained running across the five blocks within a part, but a different file was opened at each block boundary. Each material was measured in a separately launched Blender process, preventing shader or texture state from being carried between different materials. The two parts of each material were also run as separate Blender processes on different occasions.

Preliminary runs conducted during protocol development indicated that absolute render times could shift with changes in machine state and background GPU activity. The analysis therefore focuses on within-part differences between procedural and bitmap representations rather than on absolute timing differences across separate sessions. Absolute render times are reported separately for each part, whereas the representation effect is estimated within each part before the two part-level estimates are combined. This preserves the paired within-session comparison while avoiding the assumption that absolute render times measured on different occasions are directly interchangeable.

### 2.9. Variables and Analysis

The independent variable is the texture representation (procedural node graph versus baked $2048^{2}$ bitmap); the secondary factor is material, treated as four nominal test conditions, three of which are ordered by procedural computational demand. The primary dependent variable is pure sampling time. Secondary variables are the Cycles-reported internal peak memory and the deterministic image-texture footprint. Controlled variables are the Blender version, all Cycle settings, geometry, lighting, camera, render resolution, and hardware.

The nine renders within a block are consecutive and share the machine state of that block, so individual renders were not treated as independent observations. The unit of inferential analysis was therefore the block mean. For each material, a separate block-level ordinary least-squares model was fitted to the ten block means obtained across the two order-balanced parts. Mean pure sampling time was the dependent variable, and the model included texture representation (procedural or bitmap), the experimental part, and centered block position (block number minus 3) as fixed effects. The experimental part was included to account for shifts in absolute render time between sessions, while block position was included to account for possible monotonic within-part drift. The coefficient for texture representation therefore estimates the bitmap-minus-procedural render-time difference after adjustment for these two design factors.

Because each material contributed only ten block-level observations and equal residual variance could not be assumed, HC3 heteroskedasticity-robust standard errors were used. Two-sided *t* tests and 95% confidence intervals were calculated for the representation coefficient using the model residual degrees of freedom. The four primary material-specific representation tests were adjusted for multiple comparisons using the Holm procedure. The targeted $4096^{2}$ multifractal follow-up was analyzed separately using the same block-level model specification; because it comprised a single follow-up representation comparison, its two-sided *p* value is reported without multiplicity adjustment together with its 95% confidence interval. Statistical significance was assessed at α=0.05, but the absolute difference in seconds and the percentage difference relative to the procedural representation were retained as the primary measures of practical effect magnitude. Individual render measurements were used to calculate block means and descriptive within-part variability only and were not treated as independent replicates.

The material factor is treated as four separate tested conditions rather than as a continuous measure of complexity. Three of the four form an ordering by computational demand, but they differ in algorithm, coordinate system, and spatial structure as well, so the factor is nominal, and the spacing between its levels is not a measured quantity.

### 2.10. Use of Generative AI

Generative AI tools were used as assistive tools during manuscript preparation and revision. Claude (Anthropic, San Francisco, CA, USA, Claude Opus 5) assisted with drafting and debugging measurement, analysis, and plotting scripts, aggregation of the original raw logs, computation of image-comparison metrics, and language editing. ChatGPT (OpenAI, GPT-5.4) was used during revision to assist with block-level statistical analysis, revision of manuscript text, and generation of an updated statistical figure from the authors’ measured data. All scripts and statistical calculations were executed or independently checked against the raw measurement logs by the authors, and all numerical results were verified before inclusion in the manuscript. Generative AI was not used to generate measurement data, select or modify experimental observations, or design the experiment. All experimental decisions, reported measurements, interpretations, and final manuscript content remain the responsibility of the authors.

## 3. Results

### 3.1. Render Time

Table 5 summarizes the render-time results for each material. Absolute procedural and bitmap means are reported separately for the two experimental parts because the sessions were conducted on different occasions and exhibited shifts in absolute render time. Inferential results are based on the block-level models described in Section 2.9. The adjusted time difference is defined as the bitmap-minus-procedural representation coefficient, so negative values indicate that the bitmap representation was faster. The table reports HC3-based 95% confidence intervals for this coefficient and Holm-adjusted two-sided *p* values across the four primary material-specific tests. The percentage speedup is retained as a descriptive measure of practical effect magnitude and is positive when the bitmap representation was faster.

For the checker, the adjusted bitmap-minus-procedural difference was +0.081 s (95% CI: +0.040 to +0.122 s; Holm-adjusted p=0.0028), corresponding descriptively to a 0.65% advantage for the procedural representation. For noise, the adjusted difference was −0.216 s (95% CI: −0.242 to −0.191 s; Holm-adjusted p<0.001), corresponding to a 1.73% bitmap advantage. For multifractal at $2048^{2}$, the adjusted difference was −0.460 s (95% CI: −0.481 to −0.439 s; Holm-adjusted p<0.001), corresponding to a 3.53% bitmap advantage. Ambient occlusion showed a substantially larger adjusted difference of −26.630 s (95% CI: −27.222 to −26.039 s; Holm-adjusted p<0.001), corresponding to a 67.74% bitmap advantage under the tested AO configuration. All four confidence intervals excluded zero. Nevertheless, the three non-AO effects remained below 4% in relative magnitude and should therefore be distinguished from the much larger AO effect when considering practical significance.

The two parts reproduced the relative direction of the procedural–bitmap differences despite shifts in absolute render time between sessions. Figure 4 shows the descriptive part-level means on a common vertical axis beginning at zero. Figure 5 instead summarizes the block-level inferential results for the three non-AO materials as adjusted bitmap-minus-procedural coefficients with HC3-based 95% confidence intervals. Negative values indicate faster bitmap rendering, whereas positive values indicate faster procedural rendering. All three confidence intervals exclude zero, although the corresponding practical effects remain small in relative magnitude, below 4%.

A targeted follow-up experiment examined whether the multifractal bitmap remained faster when the bake resolution was increased from $2048^{2}$ to $4096^{2}$. As in the primary experiment, the procedural and bitmap branches were measured in two opposite order-balanced parts, and the procedural branch was re-measured within the follow-up sessions. At $4096^{2}$, the procedural mean was 12.992 s in Part 1 and 13.179 s in Part 2, while the bitmap mean was 12.760 s and 12.960 s, respectively. The block-level model estimated an adjusted bitmap-minus-procedural difference of −0.226 s (95% CI: −0.253 to −0.198 s; two-sided p<0.001), corresponding descriptively to a 1.73% bitmap advantage. The direction of the effect was therefore retained at the higher bake resolution, but its magnitude was smaller than the 3.53% advantage measured for the $2048^{2}$ multifractal bitmap.

The higher-resolution representation improved SSIM from 0.886 to 0.936, while increasing the image-texture footprint from 16 MiB to 64 MiB. The measured bake time also increased from 6.70 s at $2048^{2}$ to 27.842 s at $4096^{2}$. Table 6 summarizes this performance–fidelity–memory trade-off.

GPU temperature and core clock were logged for every render in the primary experiment. Across its 480 renders, the recorded temperature ranged from 42 °C to 60 °C, with a mean of 56.1 °C, while the reported core clock ranged from 1809 to 1835 MHz and assumed only three distinct values. These end-of-render observations provide no indication of sustained thermal throttling. Because temperature and clock were sampled only once at the end of each render, however, they do not characterize the complete render interval and cannot exclude brief fluctuations between measurements.

### 3.2. Block-Level Consistency

Table 7 reports the mean pure sampling time for each block in both experimental parts. Because Part 1 begins with the procedural condition and Part 2 begins with the bitmap condition, odd-numbered blocks correspond to procedural renders in Part 1 and bitmap renders in Part 2. For the checker, every procedural block mean was lower than every bitmap block mean within the corresponding part. The opposite direction was observed for noise, multifractal, and ambient occlusion, for which every bitmap block mean was lower than every procedural block mean within each part. The same direction was therefore observed under both block orders. Across the eight primary-experiment sessions, block means within each representation remained within 1% of their corresponding session mean; the largest spread was 0.57% for the procedural checker condition in Part 1. Within-part block-mean ranges did not overlap between representations. The separation between the highest block mean of the faster representation and the lowest block mean of the slower representation ranged from 0.035 s for the checker in Part 1 to 27.0 s for ambient occlusion in Part 1. Within-block standard deviations ranged from 0.017 s to 0.081 s. Figure 6 plots every render in execution order and shows the five-block structure, the three excluded warm-up renders at the beginning of each block, and the reversal of procedural–bitmap order between the two experimental parts.

### 3.3. Memory Footprint

Memory behavior was identical across all four materials, both parts and every render. Cycles-reported internal peak memory, as defined in Section 2.7, was exactly 433 MB for every procedural render and exactly 449 MB for every bitmap render, a constant difference of 16 MiB. The same two values occurred in all 480 renders without exception. This 16 MiB increment corresponds exactly to the calculated image-texture footprint: 0 MiB in the procedural branch and 16 MiB in the bitmap branch, equal to the uncompressed size of one $2048^{2}$ RGBA8 buffer (2048×2048×4 bytes = 16 MiB). This correspondence concerns the Cycles internal memory metric and should not be interpreted as a direct measurement of a 16 MiB increase in physical GPU VRAM. The maps are stored on disk as 8-bit PNG files without an alpha channel, but Blender represents them as four-channel buffers in memory, which is why the calculated footprint uses four bytes per pixel rather than three.

On disk the baked maps vary strongly with pattern content, from 0.19 MB for the checker to 5.48 MB for the multifractal, because PNG compression exploits pattern regularity (Table 8). That table separates three quantities that are easily conflated. The first column is the size of the PNG file on disk. The second is the theoretical uncompressed RGBA8 footprint of one map, a calculated figure rather than a measurement. The third and fourth are Cycles-reported internal peak memory as defined in Section 2.7, that is, Cycles’ own internal accounting rather than system RAM or GPU VRAM; every value in them was constant across all renders of every cell. Only the disk column is totaled. The four maps are never loaded together, because each material lives in its own scene file with a single material slot, so a total of the memory columns would describe a configuration that was never rendered. That variation disappears in memory, where every map of a given resolution occupies the same 16 MiB regardless of content, because the uncompressed buffer size depends only on the pixel count and the channel format. The scene files themselves are essentially identical in size across branches, between 432.9 and 433.6 KB, since the procedural material is contained entirely within the node graph stored in the .blend file, while the bitmap branch stores only a reference to an external image.

To test how that footprint scales, every material was baked again at 512, 1024, and 4096 using the identical protocol. Table 9 reports the measured PNG file sizes, and Table 10 sets the theoretical uncompressed footprint of one map beside the Cycles-reported internal peak memory measured at each resolution. On disk, the totals rise from 0.83 MB at 512 to 33.60 MB at 4096, a factor of about 40 across a sixty-fourfold increase in pixel count, with the exact factor depending on how compressible each pattern is. The memory figures are not inferred: the bitmap branch of every material was rendered again with each of the four maps swapped in, and the Cycles-reported internal peak memory was recorded. Peak memory rose from 434 MB at 512 to 437 MB at 1024, 449 MB at 2048, and 497 MB at 4096, against 433 MB for the procedural branch, so the increment over the procedural baseline is 1, 4, 16, and 64 MiB. Those increments match the theoretical uncompressed RGBA8 footprint of a single map exactly at all four resolutions, and were identical for all four materials. The procedural definitions are unchanged by resolution and remain at a few kilobytes inside the .blend file.

Table 9 and Table 10 together make the asymmetry concrete. Compressed on disk, the four maps together occupy less than 34 MB even at 4096, and a trivial pattern such as the checker costs almost nothing. Loaded for rendering, each of those same maps adds an identical 64 MiB at that resolution, whatever it depicts. The four are never loaded together here, so 256 MiB is an arithmetic extrapolation rather than a measurement, but it is the figure a scene combining four such materials would have to budget for.

## 4. Discussion

### 4.1. Render-Time Differences Across the Tested Shader Graphs

Across the three tested shader graphs, the bitmap advantage increased along the predefined ordering by computational demand, which is consistent with the pattern proposed in H2. The block-level analysis indicated representation effects distinguishable from zero for all three texture-pattern comparisons after Holm correction: checker, +0.081 s (95% CI: +0.040 to +0.122 s; adjusted p=0.0028); noise, −0.216 s (95% CI: −0.242 to −0.191 s; adjusted p<0.001); and multifractal at $2048^{2}$, −0.460 s (95% CI: −0.481 to −0.439 s; adjusted p<0.001). Statistical distinguishability should not, however, be conflated with practical magnitude: the corresponding relative differences were only 0.65%, 1.73%, and 3.53%, respectively. Nor should the observed ordering be interpreted as evidence that computational demand alone caused the trend. The checker, noise, and multifractal conditions also differ in algorithm, coordinate system, and spatial structure, and these factors were not experimentally separated. The measured result is therefore an association across the three specific tested graphs rather than an isolated complexity effect. A change in direction was observed between the tested checker and noise conditions, but the present design does not identify a general crossover point or establish a monotonic causal relationship between shader complexity and the benefit of baking. What the measurements add to the computation–storage trade-off described in previous work [9,10] is controlled evidence that representation performance differed across three practically distinct procedural graphs under the same rendering configuration. The magnitudes should also be read in a practical context. Across the three non-AO materials, the observed differences were small, remaining below 4%. For the checker material in particular, the absolute difference was less than one tenth of a second per frame. The multifractal follow-up further shows that the apparent performance benefit of baking depends on the fidelity target: increasing the bake resolution from $2048^{2}$ to $4096^{2}$ improved SSIM from 0.886 to 0.936, but reduced the bitmap render-time advantage from 3.53% to 1.73% and increased the image-texture footprint from 16 MiB to 64 MiB. The higher-resolution bitmap therefore provided better visual agreement at the cost of greater memory use and a smaller timing benefit. The practical conclusion is not that baking necessarily yields substantial savings for ordinary texture patterns, but that render-time, memory, and fidelity must be considered jointly when selecting the representation.

### 4.2. Ambient Occlusion as a Separate Case

Ambient occlusion is a mechanistically distinct case and should not be interpreted as a fourth point on the same procedural-complexity continuum as checker, noise, and multifractal. The AO node performs scene-dependent ray queries at each shading point, so its cost depends on ray traversal through the scene acceleration structure rather than only on arithmetic evaluation of a texture pattern. Under the tested configuration, baking replaced these repeated AO evaluations with bitmap lookup and reduced render time by 67.74%. This value is specific to the present scene geometry and AO settings, namely 16 samples and Distance = 1.0, and should not be extrapolated to other AO sample counts, distances, scenes, or object configurations. Additional AO settings were not tested, so the present experiment does not establish how the magnitude of the benefit scales with AO computational cost. Notably, the baked AO branch rendered at essentially the same cost as the baked branches of the other materials. Among checker, multifractal, and ambient occlusion, whose part 2 batches were measured consecutively in one uninterrupted run, the baked means spanned 0.076 s, whereas the procedural means spanned 26.3 s. Because the eight sessions were not all measured on the same occasion and absolute times differed between occasions, this comparison is restricted to conditions measured within the same uninterrupted run. Once the AO result is represented as a bitmap, the runtime cost of its original ray-traced generation is no longer evaluated during rendering. The saving reported above is a per-render saving and does not include the one-off cost of producing the baked map. Baking each material at $2048^{2}$ under the reference protocol was timed separately: 6.11 s for checker, 6.38 s for noise, 6.70 s for multifractal, and 35.83 s for ambient occlusion. Bake time is subject to the same machine-state dependence as render time; three repeat bakes of the ambient-occlusion map performed on a later occasion ranged from 38.5 s to 41.1 s. Under the measured configuration, comparing the one-off bake cost with the observed per-frame saving gives an approximate break-even point of two frames for ambient occlusion, about 15 frames for multifractal and about 30 frames for noise. These values are scenario-specific estimates rather than general break-even thresholds because both bake cost and render-time saving depend on the tested hardware, renderer configuration, and scene. For the checker, no break-even point occurred under the tested configuration because the bitmap branch was slightly slower than the procedural branch. A baked map is also a different kind of asset from the shader it replaces. It is fixed at the geometry and the surrounding scene present at bake time, so baked ambient occlusion suits static geometry and does not respond automatically to deformation, to a change of pose, or to objects moving nearby, whereas the procedural AO node re-evaluates the occlusion every frame. Where the object or its surroundings change, the map has to be rebaked, and the bake cost recurs.

These trade-offs also suggest a possible direction for adaptive or automated rendering pipelines. A future system could select between procedural evaluation and a baked representation according to the measured cost of the procedural graph, available memory budget, required visual fidelity, texture resolution, expected asset reuse, and the number of anticipated rendered frames. Such a decision could additionally account for the one-off bake cost and the need for rebaking when geometry, pose, or surrounding scene context changes. The present study does not implement or validate such an adaptive system; it only identifies measurable performance, memory, and fidelity trade-offs that could inform one.

### 4.3. Memory-Footprint Implications

Under the tested configuration, H3 is supported: the 16 MiB difference in Cycles-reported internal peak memory was constant in every render of every cell and matched the theoretical uncompressed footprint of one $2048^{2}$ RGBA8 buffer. The resolution sweep reproduced the same correspondence at $512^{2}$, $1024^{2}$ and $4096^{2}$. This agreement demonstrates how the additional baked image buffer was reflected in the internal Cycles metric under the tested configuration; it does not establish an equivalent increase in physical GPU VRAM. The asymmetry between disk storage and uncompressed image footprint is nevertheless practically relevant. PNG compression rewards pattern regularity, so the checker occupied only 0.19 MB on disk, whereas the multifractal occupied 5.48 MB, although both had the same theoretical 16 MiB uncompressed RGBA8 footprint at $2048^{2}$. Disk file size should therefore not be used as a direct proxy for the in-memory image footprint during rendering. Because the uncompressed footprint of a bitmap grows with the square of its resolution, whereas the procedural representation does not require a corresponding image buffer, the relative image-memory advantage of the procedural branch increased with texture resolution across the tested range, from 1 MiB at $512^{2}$ to 64 MiB at $4096^{2}$. Table 9 and Table 10 report both the calculated image footprint and the corresponding Cycles-reported internal memory values. These results apply to the tested configuration: one Base Color image per material, an 8-bit RGBA buffer, and the tested version of Cycles and Texture Limit disabled. Different texture-cache policies, channel formats, image types, or renderer implementations may produce different memory behavior. In scenes containing many unique high-resolution image maps, this storage trade-off may become more important than the comparatively small render-time differences observed for the three non-AO materials.

### 4.4. Context Dependence and Limitations

Taken together, the results indicate that neither representation is universally preferable. In offline rendering, a modest increase in per-frame evaluation cost may be acceptable when procedural representation provides resolution independence and avoids additional image-texture storage. In performance-constrained rendering contexts, precomputed representations may instead be advantageous when repeated procedural or ray-based evaluation becomes comparatively expensive [12,13]. The present measurements therefore support a representation-specific trade-off rather than a general rule that either procedural or baked textures are preferable. Several limitations qualify these conclusions. The primary measurements were obtained with a single renderer version, the CUDA compute backend, pre-RTX Pascal-generation GPU, and a deliberately simple test scene. Consequently, both the absolute render times and the observed procedural-versus-baked differences should be interpreted as specific to the tested Blender Cycles configuration. GPU architectures differ in memory bandwidth, cache organization, texture-sampling behavior, and ray-tracing capabilities, while renderer backends may use those hardware resources differently. These factors can alter the relative costs of procedural shader evaluation, bitmap access, and scene-dependent ray queries and may therefore change both the magnitude and, for sufficiently small effects, potentially the direction of the measured differences. The primary GTX 1050 Ti experiment consequently provides controlled within-configuration evidence rather than a universal performance ranking of procedural and baked representations. No cross-hardware replication on a contemporary RTX-class GPU was performed, so the external validity of the measured effects across newer GPU architectures and alternative Cycles backends remains untested. Only three procedural texture graphs were tested. They establish an ordering rather than a demonstrated monotonic relationship with computational demand, because they differ in algorithm, coordinate system, and spatial structure as well as in arithmetic cost, and three points do not densely sample any of those dimensions.

Each material was measured in two opposite, order-balanced parts. The direction of the procedural–bitmap difference was reproduced in both parts for every material, and the two part-level percentage estimates differed by no more than 0.04 percentage points. This agreement provides a descriptive robustness check on the within-configuration result, but it does not by itself establish generalizability beyond the tested hardware, renderer, and scene. The limitations that remain are also those of scope. The BSDF is deliberately simplified to a near-Lambertian response; only the Base Color channel is compared, with no normal, roughness, or displacement map; and the procedural noise and multifractal materials sample Generated object space while every baked map is sampled in UV space. More broadly, the perceived realism and fidelity of rendered imagery depend on multiple interacting visual cues beyond texture representation alone [35]; the present experiment deliberately isolates only one narrow representation-level trade-off. The overall economics of baking are also only partly assessed: Section 4.2 sets the measured 2048 bake times against the per-frame savings to give break-even frame counts, but those counts assume the object and its surroundings do not change, and no attempt is made to cost rebaking under animation or to account for the artist time a bake step adds to a pipeline. Finally, the alternating, order-balanced block design reduces confounding from monotonic changes in machine state and counterbalances the first-tested representation, but it does not isolate all cache-related, driver-related or other within-block warm-up effects. The three initial renders of each block were therefore treated as warm-up observations and excluded from the reported analyses, although residual short-term machine-state variation cannot be ruled out completely.

## 5. Conclusions

This study compared procedural shader graphs with their baked bitmap counterparts in Blender Cycles under controlled conditions in which geometry, camera, lighting, sampling, path depth, and BSDF parameters were held constant within each material comparison. The primary experiment comprised 480 renders, of which 360 were analyzed, and each material was evaluated in two opposite, order-balanced parts. Formal inference was conducted at the block level rather than by treating individual renders as independent replicates. The adjusted procedural–bitmap differences were statistically distinguishable from zero for all four primary materials after Holm correction, although the practical magnitude of the three non-AO effects remained below 4%. The procedural checker was 0.65% faster, whereas the bitmap branch was 1.73% faster for noise and 3.53% faster for the $2048^{2}$ multifractal representation. Although the bitmap advantage increased along the predefined ordering by computational demand, the experiment does not establish computational demand as the sole cause of this pattern because the three graphs also differ in algorithm, coordinate system, and spatial structure. Ambient occlusion was analyzed separately because it performs scene-dependent ray evaluations rather than only evaluating a texture pattern; under the tested AO configuration, baking reduced render time by 26.63 s, or 67.74%. The targeted multifractal follow-up clarified the fidelity limitation of the primary $2048^{2}$ comparison. Repeating the same order-balanced timing protocol with a $4096^{2}$ baked multifractal map improved SSIM from 0.886 to 0.936, while the bitmap branch remained faster, with an adjusted render-time difference of −0.226 s (95% CI: −0.253 to −0.198 s; p<0.001), corresponding to a 1.73% bitmap advantage. This higher-fidelity representation required a 64 MiB image-texture footprint rather than 16 MiB at $2048^{2}$, and its measured bake time increased from 6.70 s to 27.842 s. The follow-up therefore demonstrates directly that improving baked fidelity can reduce the render-time advantage while increasing both memory use and preprocessing cost. The memory result was constant under the tested configuration: throughout the 480 renders of the primary experiment, Cycles-reported internal peak memory was 433 MB for every procedural render and 449 MB for every bitmap render, giving a constant branch difference of 16 MiB that matched the calculated footprint of one uncompressed $2048^{2}$ RGBA8 buffer. The procedural branch required no image-texture footprint. Re-rendering with maps baked at $512^{2}$, $1024^{2}$, and $4096^{2}$ produced internal-metric increments of 1, 4, and 64 MiB, respectively, matching the calculated uncompressed image footprint at each resolution. These values describe Cycles’ internal memory accounting and should not be interpreted as direct measurements of physical GPU VRAM consumption. These figures apply to a single Base Color map in an 8-bit RGBA buffer, in the tested version and configuration of Cycles, with Texture Limit off. More generally, the performance differences reported in this study should not be interpreted as universal rankings of procedural and baked representations. The primary experiment used a CUDA-based GTX 1050 Ti configuration without dedicated RTX-class ray-tracing hardware, and differences in GPU architecture, memory bandwidth, cache behavior, compute backend, renderer implementation, and scene complexity may alter both the magnitude and, particularly for the small non-AO effects, potentially the direction of the observed differences. The practical implication is that the choice between procedural and baked representation should be made at the level of the individual effect rather than assumed for an entire project. Under the tested configuration, baking provided little benefit for the checker pattern, modest render-time savings for noise and multifractal, and a substantially larger saving for the ray-traced ambient-occlusion case. The overall trade-off also depends on the one-off bake cost, the expected number of rendered frames, texture resolution, memory requirements, and whether geometry or surrounding scene context changes. Using the measured $2048^{2}$ bake times, the approximate break-even points were two frames for ambient occlusion, 15 for multifractal, and 30 for noise, whereas checker did not reach a break-even point because its bitmap representation was slightly slower. The measured trade-offs may also inform future rendering systems that select dynamically between procedural and baked representations according to evaluation cost, memory availability, fidelity requirements, and expected asset reuse, although such an adaptive selection strategy remains outside the scope of the present study. Future work should replicate the experiment on contemporary RTX-class hardware and alternative Cycles backends, examine more complex scenes and materials, and determine how performance–fidelity trade-offs evolve across a broader range of bake resolutions and procedural effects.

## Figures and Tables

**Figure 1:**
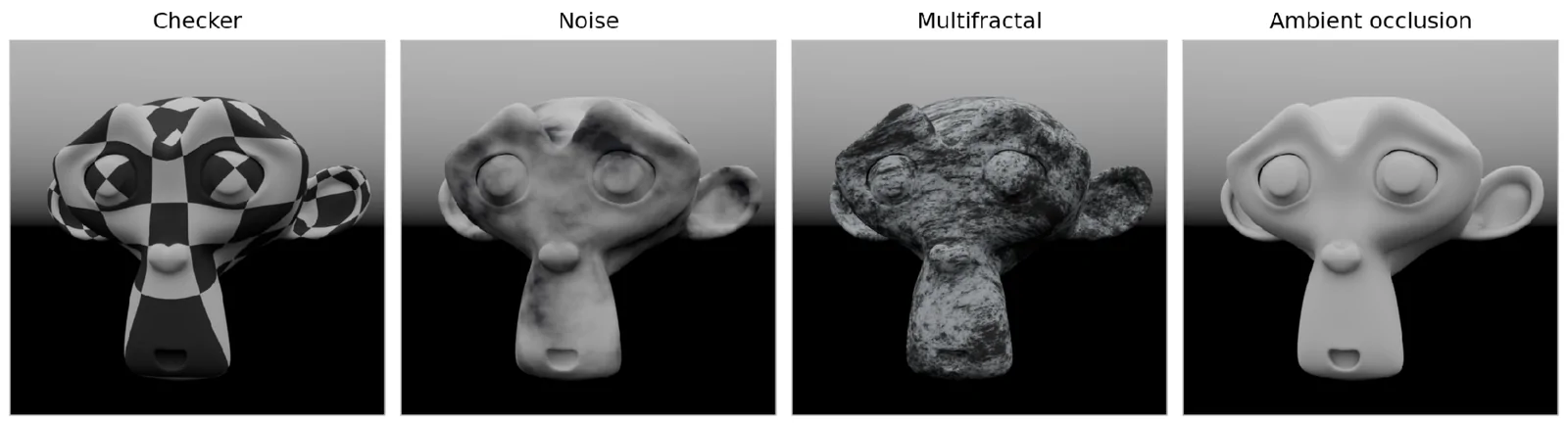
The four test materials are rendered on the common test object under the reference scene configuration. From left to right: checker, noise, multifractal, and ambient occlusion. The background is retained to show the fixed scene context used throughout the experiment.

**Figure 2:**
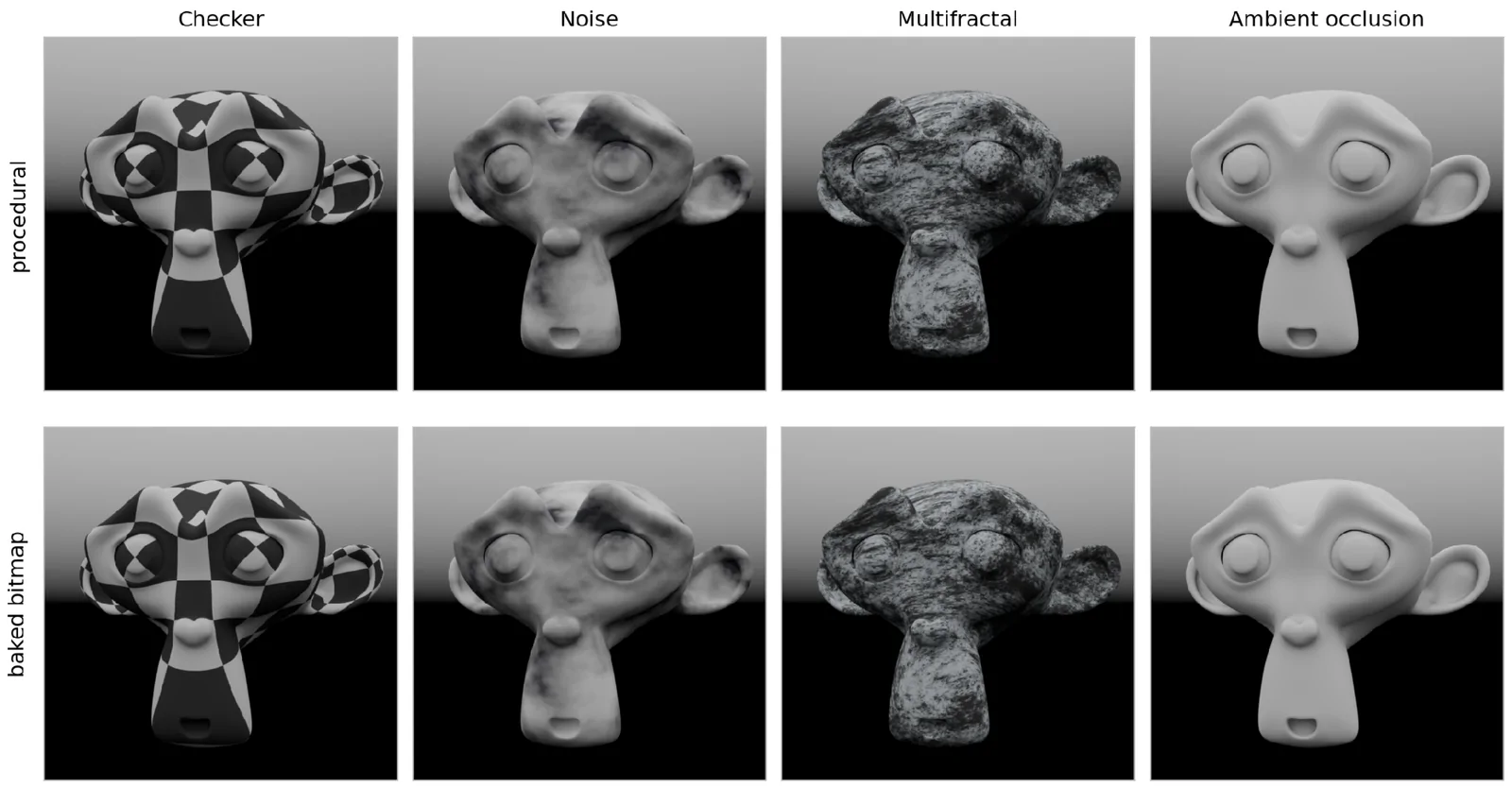
Procedural representations (**top row**) and their corresponding $2048^{2}$ baked bitmap representations (**bottom row**), rendered with the same geometry, camera, lighting, sampling, and BSDF settings.

**Figure 3:**
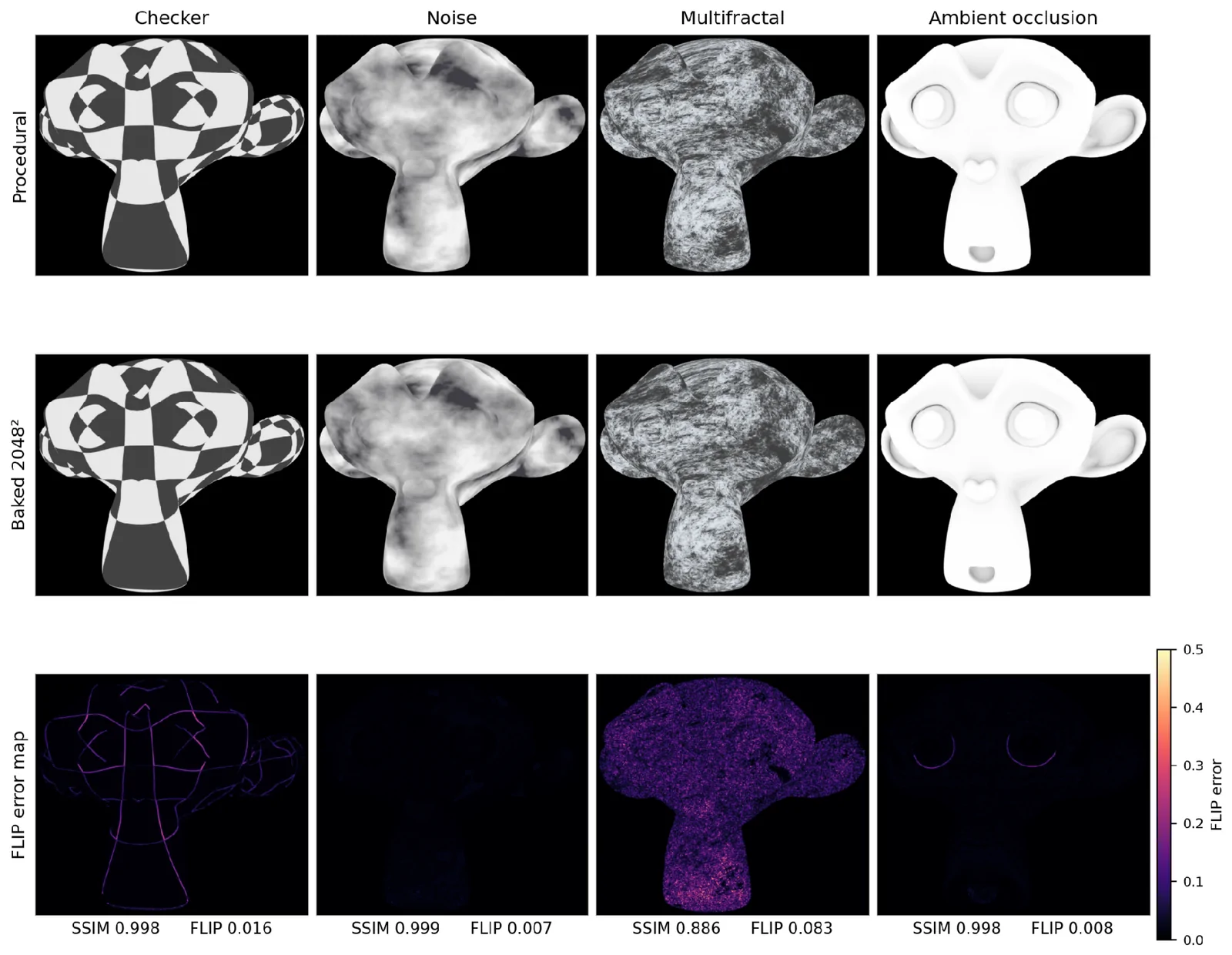
Procedural albedo (**top row**), corresponding $2048^{2}$ baked albedo (**middle row**), and FLIP error map (**bottom row**) for each material. All FLIP maps use the same 0–0.5 display range. SSIM and mean FLIP values computed over the rendered object are reported beneath each material.

**Figure 4:**
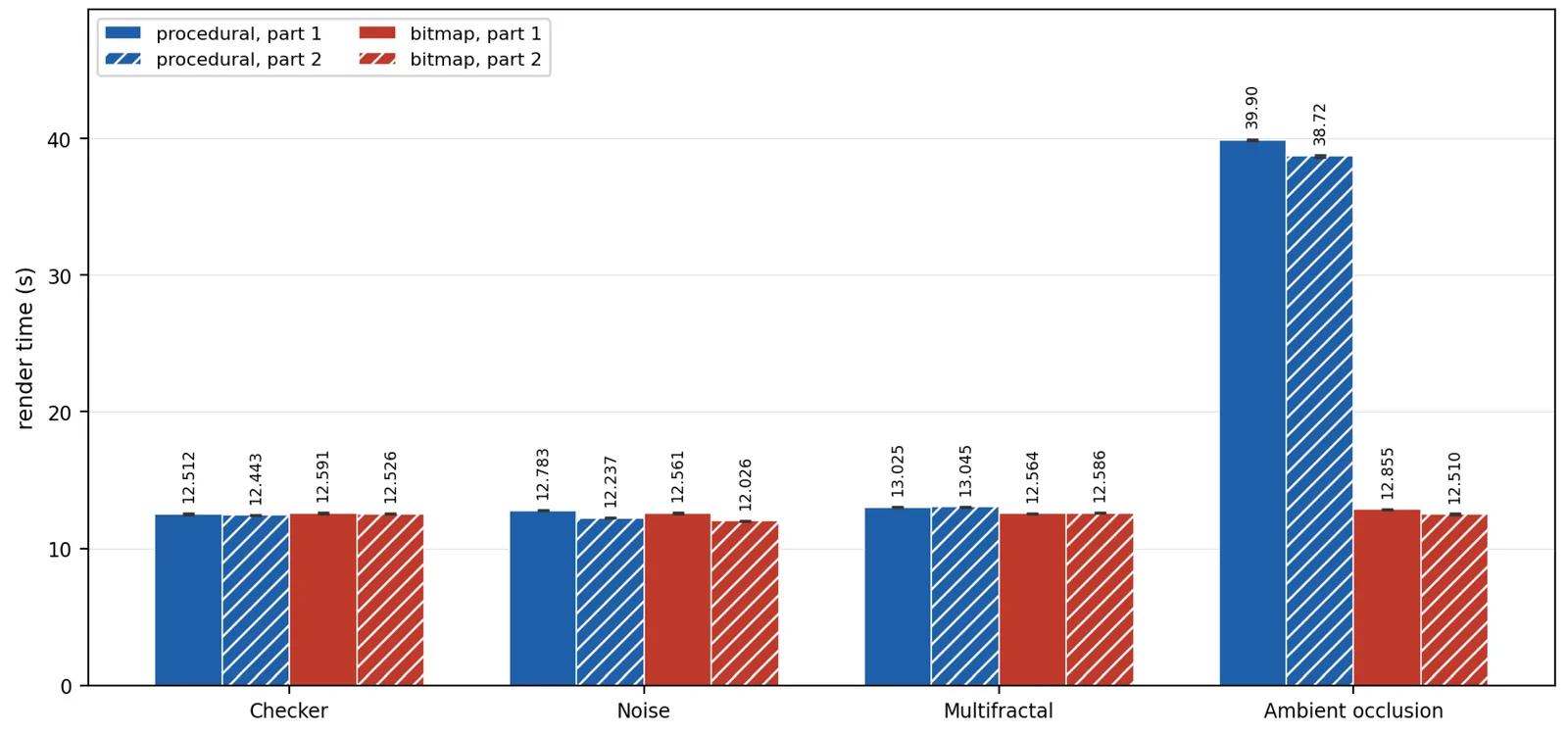
Mean pure sampling time by material, representation, and experimental part. The vertical axis begins at zero. Solid bars denote Part 1 and hatched bars denote Part 2. Error bars show one within-part standard deviation and are descriptive measures of render-to-render variability rather than confidence intervals for the procedural–bitmap effect.

**Figure 5:**
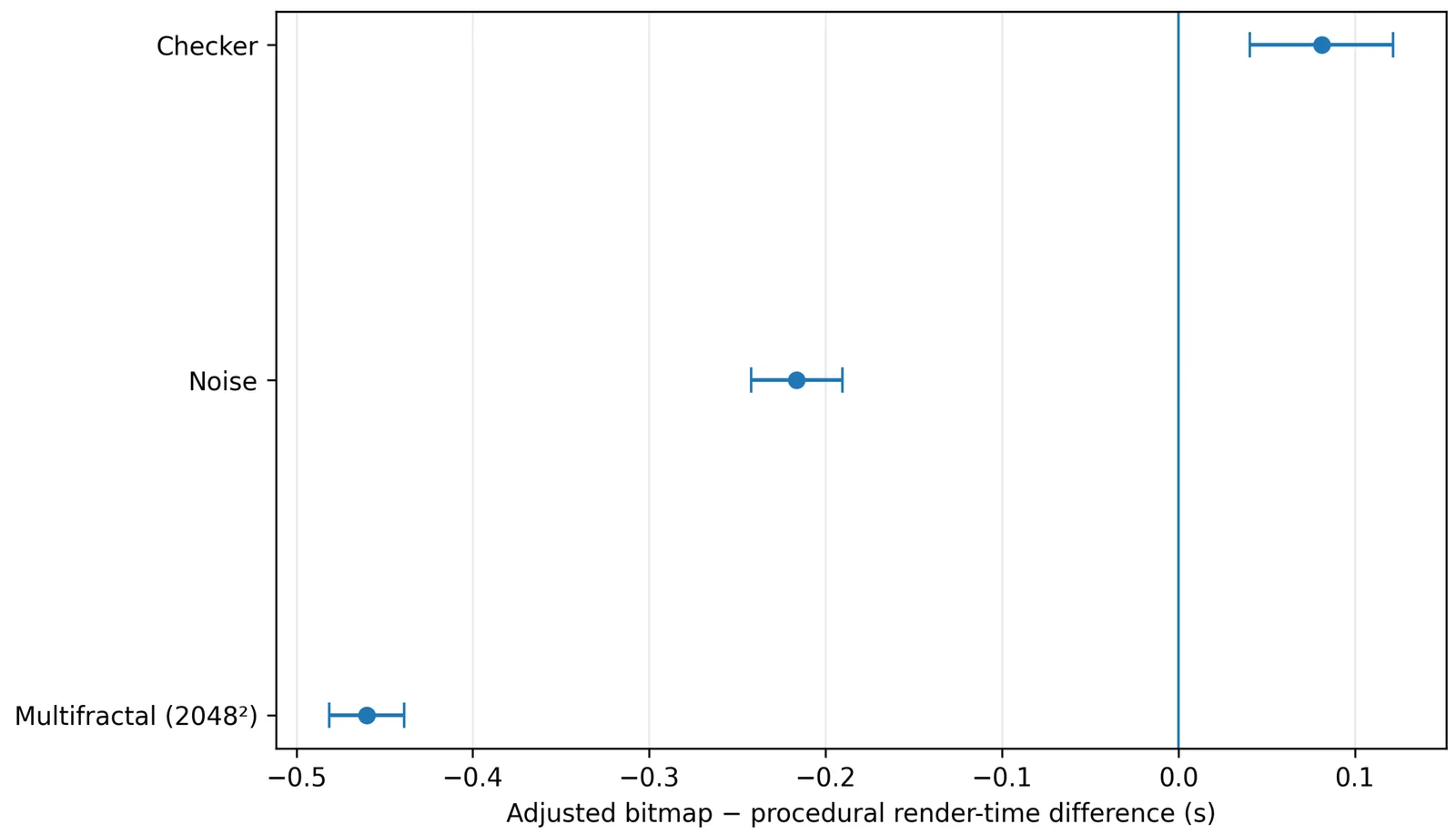
Adjusted bitmap-minus-procedural render-time differences for the three non-AO materials from the block-level models described in Section 2.9. Points show the estimated representation coefficient, and horizontal error bars show HC3-based 95% confidence intervals. Negative values indicate faster bitmap rendering, whereas positive values indicate faster procedural rendering. Ambient occlusion is omitted from this figure because its substantially larger effect would compress the scale of the three texture-pattern comparisons.

**Figure 6:**
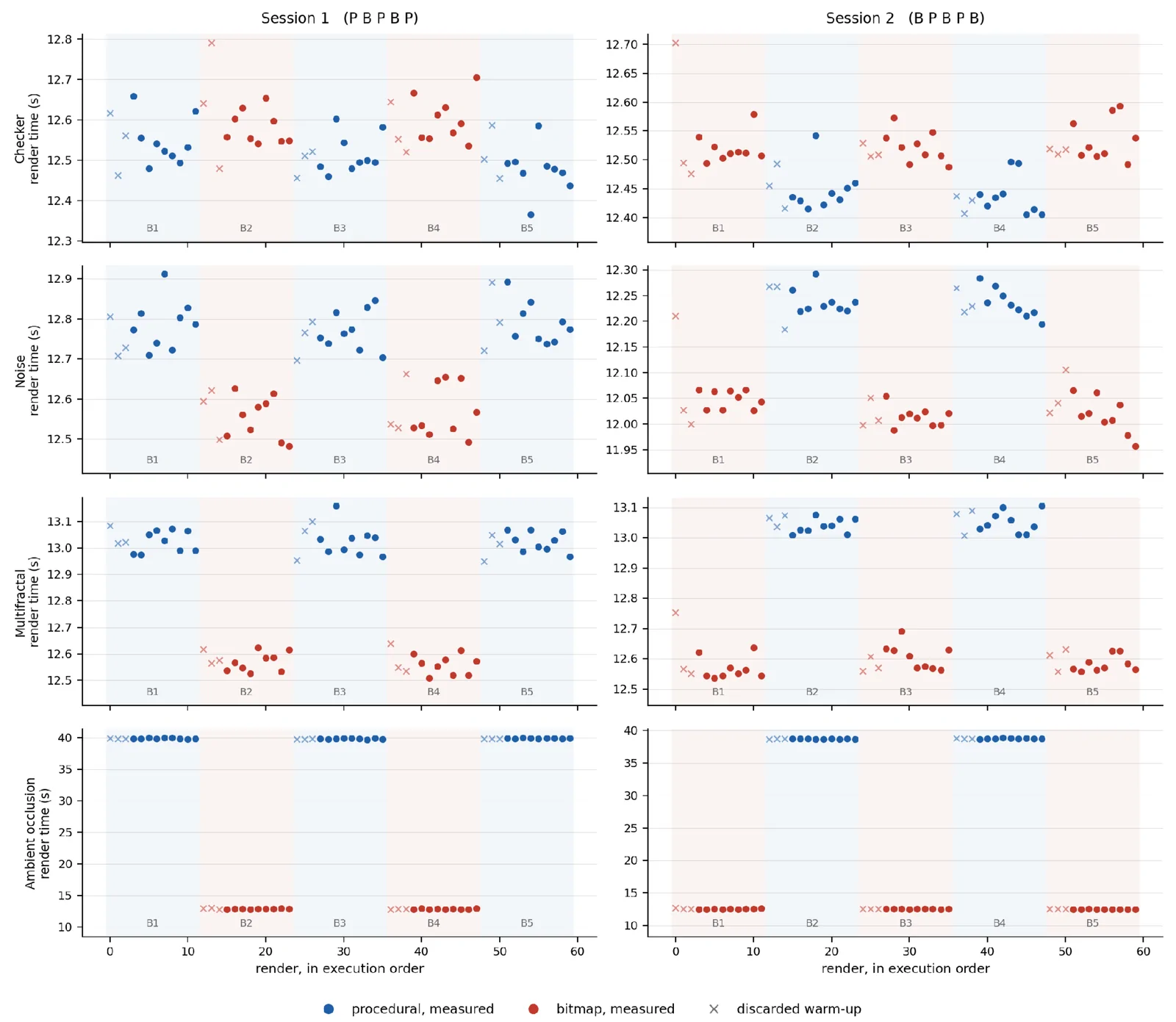
Pure sampling time for all renders in execution order, with materials arranged by row and experimental parts by column. Filled circles denote analyzed renders and crosses denote excluded warm-up renders. Shaded regions indicate blocks B1–B5 and distinguish the procedural and bitmap conditions. Part 1 begins with the procedural condition, whereas Part 2 begins with the bitmap condition. Vertical-axis limits differ between panels to preserve visibility of within-material variation.

**Table 1:** Hardware and software configuration used for the primary GTX 1050 Ti benchmark.

Component	Specification
CPU	Intel Core i5-7400 (Intel Corporation, Santa Clara, CA, USA), 4 cores/4 threads, 3.00 GHz
System memory	16 GB DDR4, 2×8 GB at 2400 MT/s
GPU	GIGABYTE NVIDIA GeForce GTX 1050 Ti (GIGA-BYTE Technology Co., Ltd., New Taipei City, Taiwan), 4 GB GDDR5, PCIe 3.0×16
GPU driver	582.28, CUDA 13.0
Compute backend	CUDA
Renderer	Blender 5.2.0 LTS, build fbe6228777e7, Cycles
Operating system	Windows 10 Home, version 22H2, build 19045
Power plan	Balanced

**Table 2:** Cycles render settings held constant between the procedural and baked branches of each comparison.

Setting	Value
Samples	256
Adaptive sampling	disabled
Denoising	disabled
Resolution	1000×1000 px
Bounces (total/diffuse/glossy/ transmission/volume)	12/4/4/12/0
Minimum light bounces	12
Clamp indirect	10.0
Caustics (reflective/refractive)	enabled
Texture limit	off
Automatic tiling/tile size	enabled/2048 px
Persistent data	disabled
Pixel filter	Blackman-Harris, width 1.5 px
View transform	AgX, look None, exposure 0.0, gamma 1.0

**Table 3:** Test materials. Checker, noise, and multifractal form the predefined ordering by procedural evaluation demand; ambient occlusion is analyzed separately as a ray-traced effect.

Material	Coordinates	Node Chain and Parameters
Checker	UV	Texture Coordinate → Checker Texture, Scale 15, Color1 (0.800, 0.800, 0.800), Color2 (0.055, 0.055, 0.055)
Noise	Generated	Texture Coordinate → Noise Texture, fBM, 3D, Scale 6, Detail 8, Roughness 0.5, Lacunarity 2, Offset 0, Gain 1, Distortion 0, Normalize on → Color Ramp, linear, 0.35 → (0.050, 0.050, 0.060), 0.65 → (0.900, 0.900, 0.900)
Multifractal	Generated	Texture Coordinate → Noise Texture, Multifractal, 3D, Scale 2, Detail 15, Roughness 1, Lacunarity 2, Offset 0, Gain 1, Distortion 0.5, Normalize on → Color Ramp, linear, 0.523 → (0.050, 0.050, 0.050), 1.000 → (0.746, 0.798, 0.850)
Ambient occlusion	n/a	Ambient Occlusion, 16 samples, Distance 1.0, Inside off, Only Local off → Map Range, linear, 0–1 to 0.05–1, clamped

*Note: n/a = not applicable; the Ambient Occlusion node does not use texture coordinates.*

**Table 4:** Image-space agreement between each procedural albedo and its $2048^{2}$ baked counterpart, measured over the rendered object using SSIM and mean FLIP.

Material	SSIM	Mean FLIP
Checker	0.998	0.016
Noise	0.999	0.007
Multifractal	0.886	0.083
Ambient occlusion	0.998	0.008

**Table 5:** Pure sampling time and block-level representation effects. Absolute means are reported separately for the two experimental parts. Adjusted bitmap-minus-procedural differences were estimated from the block-level models described in Section 2.9; confidence intervals use HC3 robust standard errors, and *p* values are Holm-adjusted across the four primary material-specific tests. Percentage speedup is descriptive. Each material contributes ten block means to the inferential analysis.

Material	Procedural (s), Part 1/Part 2	Bitmap (s), Part 1/Part 2	Adjusted B − P Difference, s (95% CI)	Bitmap Speedup, %	Holm-Adjusted *p*
Checker	12.512/12.443	12.591/12.526	+0.081 [+0.040, +0.122]	−0.65	0.0028
Noise	12.783/12.237	12.561/12.026	−0.216 [−0.242, −0.191]	+1.73	<0.001
Multifractal	13.025/13.045	12.564/12.586	−0.460 [−0.481, −0.439]	+3.53	<0.001
Ambient occlusion	39.901/38.725	12.855/12.510	−26.630 [−27.222, −26.039]	+67.74	<0.001

**Table 6:** Multifractal performance–fidelity trade-off at the two tested bake resolutions. The adjusted bitmap-minus-procedural timing effects are estimated from the same block-level model specification used for the primary analysis. Negative timing differences indicate faster bitmap rendering.

Bake Resolution	SSIM	Adjusted B − P Difference, s (95% CI)	Bitmap Speedup, %	Image-Texture Increment (MiB)	Bake Time (s)
$2048^{2}$	0.886	−0.460 [−0.481, −0.439]	3.53	16	6.70
$4096^{2}$	0.936	−0.226 [−0.253, −0.198]	1.73	64	27.842

**Table 7:** Mean pure sampling time per block for each experimental part. Each block mean is based on nine analyzed renders; the three preceding warm-up renders are excluded.

Material	Part	B1	B2	B3	B4	B5
Checker	1	12.546	12.581	12.515	12.602	12.475
Checker	2	12.520	12.447	12.523	12.439	12.535
Noise	1	12.788	12.553	12.772	12.568	12.789
Noise	2	12.048	12.238	12.014	12.235	12.016
Multifractal	1	13.024	12.569	13.027	12.559	13.024
Multifractal	2	12.568	13.039	12.608	13.052	12.583
Ambient occlusion	1	39.917	12.861	39.853	12.849	39.933
Ambient occlusion	2	12.533	38.688	12.523	38.762	12.475

**Table 8:** Memory footprint per material. Cycle values denote the internal renderer memory metric defined in Section 2.7, not direct physical GPU VRAM consumption.

Material	PNG File Size on Disk (MB)	Theoretical Uncompressed RGBA8 (MiB)	Cycles Internal Peak, Procedural (MB)	Cycles Internal Peak, Bitmap (MB)
Checker	0.19	16	433	449
Noise	2.58	16	433	449
Multifractal	5.48	16	433	449
Ambient occlusion	0.88	16	433	449
Total on disk	9.12	n/a	n/a	n/a

*Note: n/a = not applicable; only the disk-size column is meaningfully totaled because the four maps were not loaded simultaneously.*

**Table 9:** Compressed PNG file size of the baked maps on disk at each tested bake resolution.

Material	$512^{2}$ (MB)	$1024^{2}$ (MB)	$2048^{2}$ (MB)	$4096^{2}$ (MB)
Checker	0.04	0.09	0.19	0.44
Noise	0.26	0.83	2.58	8.00
Multifractal	0.40	1.47	5.48	20.75
Ambient occlusion	0.12	0.37	0.88	4.41
Total on disk	0.83	2.76	9.12	33.60

**Table 10:** Theoretical uncompressed footprint of one baked map beside the Cycles-reported internal peak memory measured with that map loaded. The Cycles value is an internal renderer metric and not a direct measurement of physical GPU VRAM.

Bake Resolution	Theoretical Uncompressed RGBA8 (MiB)	Cycles Internal Peak, Procedural (MB)	Cycles Internal Peak, Bitmap (MB)	Increment (MiB)
$512^{2}$	1	433	434	1
$1024^{2}$	4	433	437	4
$2048^{2}$	16	433	449	16
$4096^{2}$	64	433	497	64

**Figure A1:**
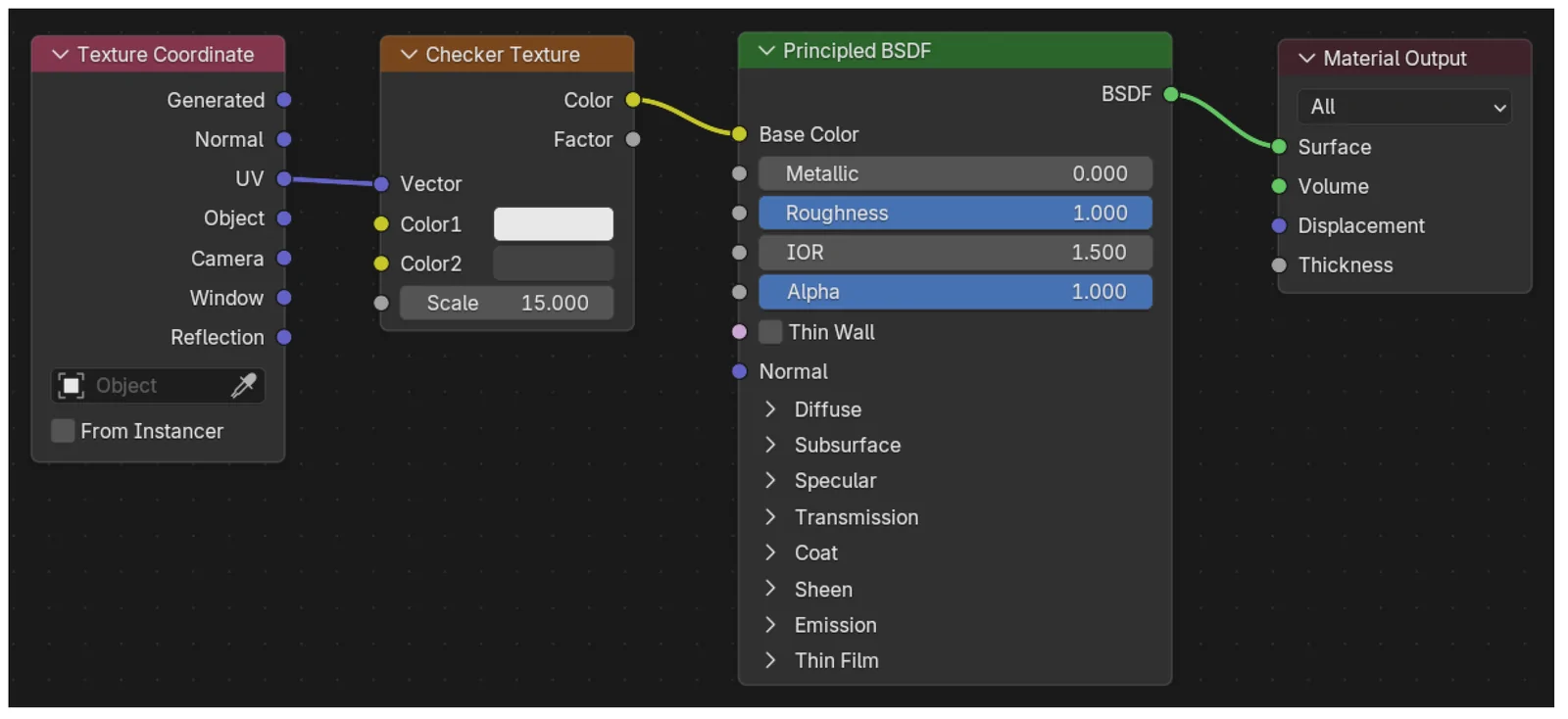
Node graph of the procedural checker material in the Blender 5.2 Shader Editor. Texture Coordinate supplies UV coordinates to a Checker Texture with Scale 15, whose Color output drives the Base Color input of the shared Principled BSDF.

**Figure A2:**
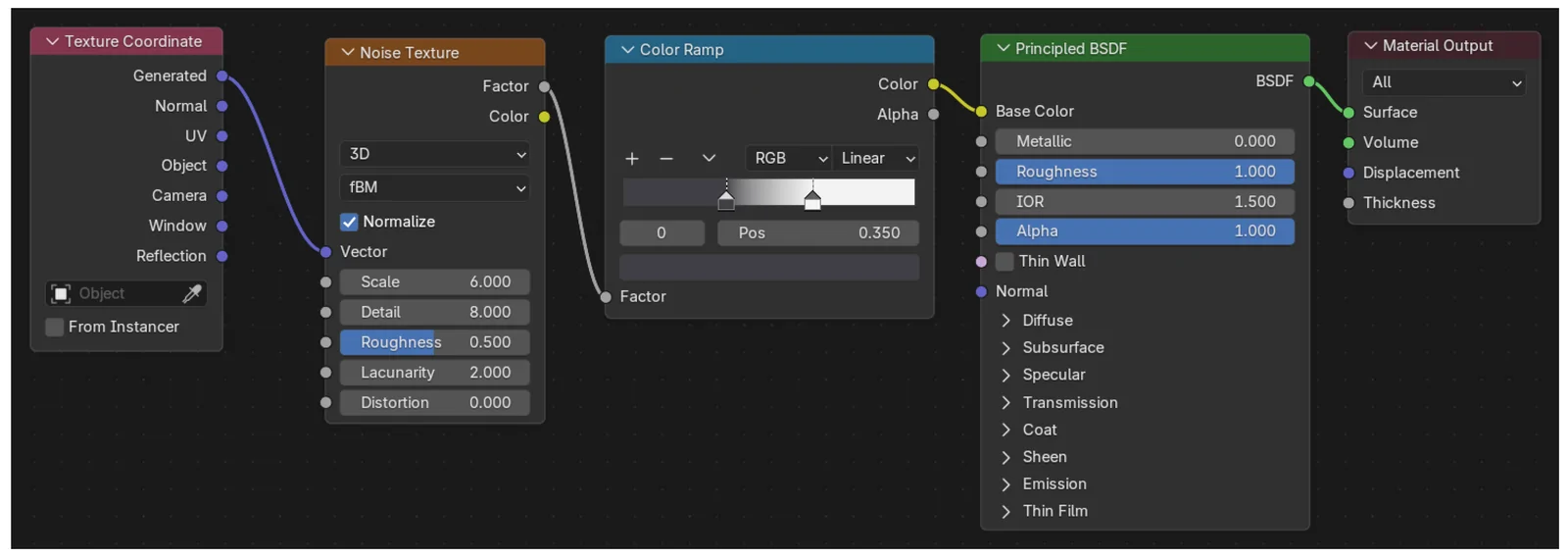
Node graph of the procedural noise material. Texture Coordinate supplies Generated coordinates to a Noise Texture of type fBM with Normalize enabled, whose Factor output drives a Color Ramp in linear mode, which in turn drives Base Color.

**Figure A3:**
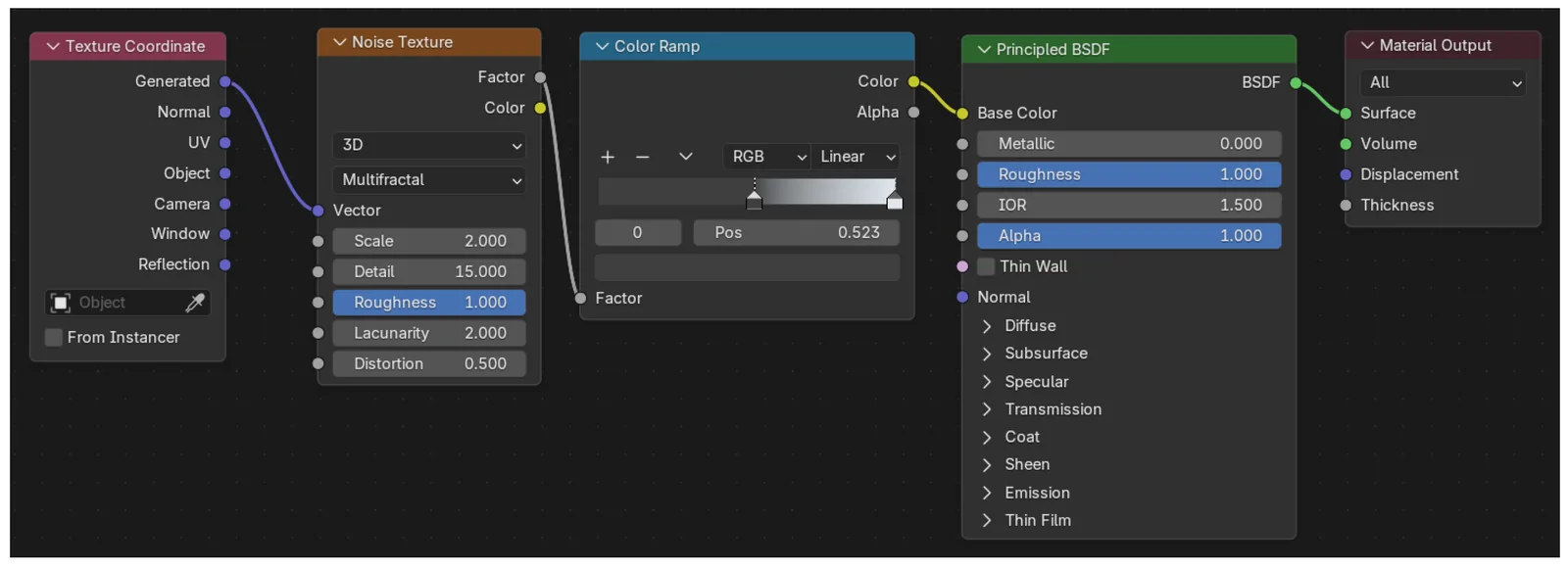
Node graph of the procedural multifractal material. The chain matches Figure A2, except that the Noise Texture is of type Multifractal with Scale 2, Detail 15, Roughness 1 and Distortion 0.5, and the Color Ramp stops differ.

**Figure A4:**
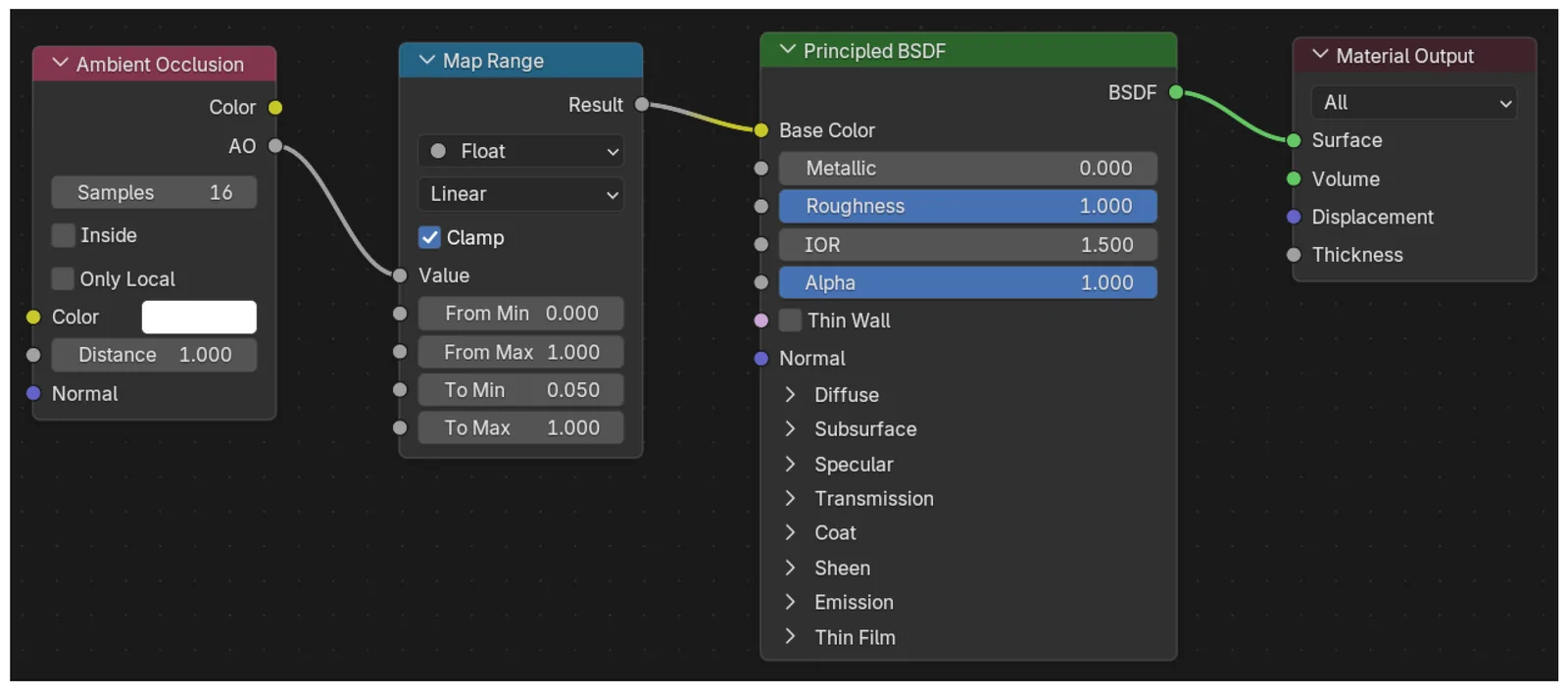
Node graph of the procedural ambient-occlusion material. The Ambient Occlusion node, at 16 samples and Distance 1.0 with Inside and Only Local disabled, feeds a Map Range node that remaps 0–1 to 0.05–1 with clamping, which drives Base Color.

## Data Availability

The materials supporting the reported results comprise the Blender scene files, embedded measurement scripts, analysis and plotting scripts, raw per-render JSON reports, cumulative tab-separated logs, block-level summaries, albedo renders and image-comparison scripts, and the baked textures at all tested resolutions. The complete raw data and supporting files for both the primary 2048^2^ experiment and the targeted 4096^2^ multifractal follow-up are available from the corresponding author upon reasonable request.

## Appendix A. Procedural Node Graphs

The four procedural node graphs described in Table 3 are reproduced here as screenshots of the Blender 5.2 Shader Editor, so that the graph structure and the parameter values can be read directly rather than reconstructed from the table. In every case the chain terminates in the Base Color input of the shared Principled BSDF, whose other inputs are identical across all eight scene files and are listed in Section 2.4. The bitmap branch of each material replaces the chain shown here with a single Image Texture node reading the baked map, with the settings given in Section 2.6.
